# Refined Young Inequality and Its Application to Divergences

**DOI:** 10.3390/e23050514

**Published:** 2021-04-23

**Authors:** Shigeru Furuichi, Nicuşor Minculete

**Affiliations:** 1 Department of Information Science, College of Humanities and Sciences, Nihon University, 3-25-40, Sakurajyousui, Setagaya-ku, Tokyo 156-8550, Japan; furuichi@chs.nihon-u.ac.jp; 2 Faculty of Mathematics and Computer Science, Transilvania University of Braşov, 500091 Brasov, Romania

**Keywords:** Young inequality, arithmetic mean, geometric mean, Heinz mean, Cartwright–Field inequality, Tsallis divergence, Rényi divergence, Jeffreys–Tsallis divergence, Jensen–Shannon–Tsallis divergence, 26D15, 26E60, 94A17

## Abstract

We give bounds on the difference between the weighted arithmetic mean and the weighted geometric mean. These imply refined Young inequalities and the reverses of the Young inequality. We also studied some properties on the difference between the weighted arithmetic mean and the weighted geometric mean. Applying the newly obtained inequalities, we show some results on the Tsallis divergence, the Rényi divergence, the Jeffreys–Tsallis divergence and the Jensen–Shannon–Tsallis divergence.

## 1. Introduction

The Young integral inequality is the source of many basic inequalities. Young [1] proved the following: suppose that $f:\left[0,\infty \right)\to \left[0,\infty \right)$ is an increasing continuous function such that $f\left(0\right)=0$ and $\lim_{x\to \infty }f\left(x\right)=\infty$. Then:

(1) $$ab\leq \int_{0}^{a}f\left(x\right)dx+\int_{0}^{b}f^{-1}\left(x\right)dx,$$

with equality if b=f(a). Such a gap is often used to define the Fenchel–Legendre divergence in information geometry [2,3]. For $f\left(x\right)=x^{p-1},\left(p>1\right)$, in inequality (1), we deduce the classical Young inequality:

(2) $$ab\leq \frac{a^{p}}{p}+\frac{b^{q}}{q},$$

for all a,b>0 and p,q>1 with $\frac{1}{p}+\frac{1}{q}=1$. The equality occurs if and only if $a^{p}=b^{q}$.

Minguzzi [4] proved a reverse Young inequality in the following way:

(3) $$0\leq \frac{a^{p}}{p}+\frac{b^{q}}{q}-ab\leq \left(b-a^{p-1}\right)\left(b^{q-1}-a\right),$$

for all a,b>0 and p,q>1 with $\frac{1}{p}+\frac{1}{q}=1$.

The classical Young inequality (2) is rewitten as

(4) $$a^{1/p}b^{1/q}\leq \frac{a}{p}+\frac{b}{q}$$

by putting $a≔a^{1/p}$ and $b≔b^{1/q}$. Putting again:

$$a≔\frac{a_{j}^{p}}{\sum_{j=1}^{n}a_{j}^{p}},\;\;\;\;b≔\frac{b_{j}^{q}}{\sum_{j=1}^{n}b_{j}^{q}}$$

in the inequality (4), we obtain the famous Hölder inequality:

$$\sum_{j=1}^{n}a_{j}b_{j}\leq {\left(\sum_{j=1}^{n}a_{j}^{p}\right)}^{1/p}{\left(\sum_{j=1}^{n}b_{j}^{q}\right)}^{1/q},\;\;\;\;\left(p,q>1,\;\;\frac{1}{p}+\frac{1}{q}=1\right)$$

for $a_{1},\cdots ,a_{n}>0$ and $b_{1},\cdots ,b_{n}>0$. Thus, the inequality (2) is often reformulated as

(5) $$a^{p}b^{1-p}\leq pa+\left(1-p\right)b,\;\;a,b>0,\;\;0\leq p\leq 1$$

by putting 1/p≕p (then 1/q=1−p) in the inequality (4). It is notable that α-divergence is related to the difference between the weighted arithmetic mean and the weighted geometric mean [5]. For p=1/2, we deduce the inequality between the geometric mean and the arithmetic mean, $G\left(a,b\right)≔\sqrt{ab}\leq \frac{a+b}{2}≕A\left(a,b\right)$. The Heinz mean ([6], Equation (3)) (see also [7]) is defined as $H_{p}\left(a,b\right)=\frac{a^{p}b^{1-p}+a^{1-p}b^{p}}{2}$ and $G\left(a,b\right)\leq H_{p}\left(a,b\right)\leq A\left(a,b\right)$.

Especially, when we discuss Young inequality, we will refer to the last form. We consider the following expression:

(6) $$d_{p}\left(a,b\right)≔pa+\left(1-p\right)b-a^{p}b^{1-p}$$

which implies that $d_{p}\left(a,b\right)\geq 0$ and $d_{p}\left(a,a\right)=d_{0}\left(a,b\right)=d_{1}\left(a,b\right)=0$. We remark the following properties:

$$d_{p}\left(a,b\right)=b\cdot d_{p}\left(\frac{a}{b},1\right),\;\;d_{p}\left(a,b\right)=d_{1-p}\left(b,a\right),\;\;d_{p}\left(\frac{1}{a},\frac{1}{b}\right)=\frac{1}{ab}\cdot d_{p}\left(b,a\right).$$

Cartwright–Field inequality (see, e.g., [8]) is often written as follows:

(7) $$\frac{1}{2}p\left(1-p\right)\frac{{\left(a-b\right)}^{2}}{\max\{a,b\}}\leq d_{p}\left(a,b\right)\leq \frac{1}{2}p\left(1-p\right)\frac{{\left(a-b\right)}^{2}}{\min\{a,b\}}$$

for a,b>0 and 0≤p≤1. This double inequality gives an improvement of the Young inequality, and at the same time, gives a reverse inequality for the Young inequality.

Kober proved in [9] a general result related to an improvement of the inequality between arithmetic and geometric means, which for n=2 implies the inequality:

(8) $$r{\left(\sqrt{a}-\sqrt{b}\right)}^{2}\leq d_{p}\left(a,b\right)\leq \left(1-r\right){\left(\sqrt{a}-\sqrt{b}\right)}^{2}$$

where a,b>0, 0≤p≤1 and $r=\min\left\{p,1-p\right\}$. This inequality was rediscovered by Kittaneh and Manasrah in [10] (See also [11]).

Finally, we found, in [12], another improvement of the Young inequality and a reverse inequality, given as

(9) $$r{\left(\sqrt{a}-\sqrt{b}\right)}^{2}+A\left(p\right)\log^{2}\left(\frac{a}{b}\right)\leq d_{p}\left(a,b\right)\leq \left(1-r\right){\left(\sqrt{a}-\sqrt{b}\right)}^{2}+B\left(p\right)\log^{2}\left(\frac{a}{b}\right)$$

where a,b≥1, 0<p<1 and $r=\min\left\{p,1-p\right\}$ with $A\left(p\right)=\frac{p\left(1-p\right)}{2}-\frac{r}{4},B\left(p\right)=\frac{p\left(1-p\right)}{2}-\frac{1-r}{4}$. It is remarkable that the inequalities (9) give a further refinement of (8), since A(p)≥0 and B(p)≤0.

In [13], we also presented two inequalities which give two different reverse inequalities for the Young inequality:

(10) $$0\leq d_{p}\left(a,b\right)\leq a^{p}b^{1-p}\exp\left\{\frac{p\left(1-p\right){\left(a-b\right)}^{2}}{\min^{2}\left\{a,b\right\}}\right\}-a^{p}b^{1-p}$$

and:

(11) $$0\leq d_{p}\left(a,b\right)\leq p\left(1-p\right)\log^{2}\left(\frac{a}{b}\right)\max\left\{a,b\right\}$$

where a,b>0, 0≤p≤1. See ([14], Chapter 2) for recent advances on refinements and reverses of the Young inequality.

The α-divergence is related to the difference of a weighted arithmetic mean with a geometric mean [5]. We mention that the gap is used in information geometry to define the Fenchel–Legendre divergence [2,3]. We give bounds on the difference between the weighted arithmetic mean and the weighted geometric mean. These imply refined Young inequalities and the reverses of the Young inequality. We also studied some properties on the difference between the weighted arithmetic mean and the weighted geometric mean. Applying the newly obtained inequalities, we show some results on the Tsallis divergence, the Rényi divergence, the Jeffreys–Tsallis divergence and the Jensen–Shannon–Tsallis divergence [15,16]. The parametric Jensen–Shannon divergence can be used to detect unusual data, and this one can also use it as a means to perform the relevant analysis of fire experiments [17].

## 2. Main Results

We give estimates on $d_{p}\left(a,b\right)$ and also study the properties of $d_{p}\left(a,b\right)$. We give the following estimates of $d_{p}\left(a,b\right)$ first.

**Theorem** **1.** *For 0<a,b≤1 and 0≤p≤1, we have:*

(12) $$r{\left(\sqrt{a}-\sqrt{b}\right)}^{2}+A\left(p\right)ab\cdot \log^{2}\left(\frac{a}{b}\right)\leq d_{p}\left(a,b\right)\leq \left(1-r\right){\left(\sqrt{a}-\sqrt{b}\right)}^{2}+B\left(p\right)ab\cdot \log^{2}\left(\frac{a}{b}\right)$$

*where $r=\min\left\{p,1-p\right\}$ and $A\left(p\right)=\frac{p\left(1-p\right)}{2}-\frac{r}{4},B\left(p\right)=\frac{p\left(1-p\right)}{2}-\frac{1-r}{4}$.*

**Proof.**  For p=0 or p=1 or a=b, we have equality. We assume a≠b and 0<p<1. Because 0<a,b≤1, we have $\frac{1}{a},\frac{1}{b}\geq 1$, so, applying inequality (9), we deduce the following relation:

(13) $$r{\left(\frac{1}{\sqrt{a}}-\frac{1}{\sqrt{b}}\right)}^{2}+A\left(p\right)\log^{2}\left(\frac{b}{a}\right)\leq d_{p}\left(\frac{1}{a},\frac{1}{b}\right)\leq \left(1-r\right){\left(\frac{1}{\sqrt{a}}-\frac{1}{\sqrt{b}}\right)}^{2}+B\left(p\right)\log^{2}\left(\frac{b}{a}\right).$$

We know that $d_{p}\left(\frac{1}{a},\frac{1}{b}\right)=\frac{1}{ab}\cdot d_{1-p}\left(a,b\right)$ and if we replace *p* by 1−p in relation (13) and because A(p)=A(1−p),B(1−p)=B(p), then we proved the inequality from the statement. □

**Theorem** **2.** *For a≥b>0 and 0<p≤1, we have:*

$$\frac{p\left(a-b\right)\left(a^{1-p}-b^{1-p}\right)}{2a^{1-p}}\leq d_{p}\left(a,b\right)\leq \frac{p\left(a-b\right)\left(a^{1-p}-b^{1-p}\right)}{a^{1-p}}.$$

**Proof.**  For p=1 or a=b, we have equality. We assume a>b and 0<p<1. It is easy to see that:

(14) $$\int_{1}^{x}\left(1-t^{p-1}\right)dt=x-1-\frac{x^{p}-1}{p}.$$

We take x=a/b in (14) and then obtain:

$$pb\int_{1}^{a/b}\left(1-t^{p-1}\right)dt=d_{p}\left(a,b\right),\;\;0<p<1$$

Then, we take the function $f:\left[1,a/b\right]\to R$ defined by $f\left(t\right)≔1-t^{p-1}$. By simple calculations we have:

$$\frac{df(t)}{dt}=\left(1-p\right)t^{p-2}\geq 0,\;\;\frac{d^{2}f\left(t\right)}{dt^{2}}=\left(1-p\right)\left(p-2\right)t^{p-3}\leq 0.$$

So the function *f* is concave so that we can apply Hermite–Hadamard inequality [18]:

$$\frac{1}{2}\left(f(1)+f(a/b)\right)\leq \frac{1}{a/b-1}\int_{1}^{a/b}\left(1-t^{p-1}\right)dt\leq f\left(\frac{1+a/b}{2}\right).$$

The left-hand side of the inequalities above shows:

$$\frac{p\left(a-b\right)\left(a^{1-p}-b^{1-p}\right)}{2a^{1-p}}\leq d_{p}\left(a,b\right).$$

Since the function $f\left(t\right)≔1-t^{1-p}$ is increasing, we have:

$$1-t^{p-1}\leq 1-x^{p-1},\;\;\left(t\leq x,\;\;0<p<1\right).$$

Integrating the above inequality by *t* from 1 to *x*, we obtain:

$$\int_{1}^{x}\left(1-t^{p-1}\right)dt\leq \left(x-1\right)\left(1-x^{p-1}\right)$$

which implies:

$$d_{p}\left(a,b\right)=bp\int_{1}^{a/b}\left(1-t^{p-1}\right)dt\leq bp\left(a/b-1\right)\left(1-{\left(a/b\right)}^{p-1}\right)=\frac{p\left(a-b\right)\left(a^{1-p}-b^{1-p}\right)}{a^{1-p}}.$$

□

**Theorem** **3.** *For a,b>0 and 0≤p≤1, we have:*

(15) $$p\left(1-p\right)\frac{{\left(a-b\right)}^{2}}{\max\{a,b\}}\leq d_{p}\left(a,b\right)+d_{1-p}\left(a,b\right)\leq p\left(1-p\right)\frac{{\left(a-b\right)}^{2}}{\min\{a,b\}}$$

**Proof.**  We give two different proofs (I) and (II).

(I) For a=b or p∈{0,1}, we obtain equality in the relation from the statement. Thus, we assume a≠b and $p\in \left(0,1\right)$. It is easy to see that $d_{p}\left(a,b\right)+d_{1-p}\left(a,b\right)=a+b-a^{p}b^{1-p}-a^{1-p}b^{p}=\left(a^{p}-b^{p}\right)\left(a^{1-p}-b^{1-p}\right)$. Using the Lagrange theorem, there exists $c_{1}$ and $c_{2}$ between *a* and *b* such that $\left(a^{p}-b^{p}\right)\left(a^{1-p}-b^{1-p}\right)=p\left(1-p\right){\left(a-b\right)}^{2}c_{1}^{p-1}c_{2}^{-p}$. However, we have the inequality $\frac{1}{\max\{a,b\}}\leq \frac{1}{c_{1}^{1-p}c_{2}^{p}}\leq \frac{1}{\min\{a,b\}}$. Therefore, we deduce the inequality of the statement.
(II) Using the Cartwright–Field inequality, we have:
  $$\frac{1}{2}p\left(1-p\right)\frac{{\left(a-b\right)}^{2}}{\max\{a,b\}}\leq d_{p}\left(a,b\right)\leq \frac{1}{2}p\left(1-p\right)\frac{{\left(a-b\right)}^{2}}{\min\{a,b\}}$$
  and if we replace *p* by 1−p, we deduce:
  $$\frac{1}{2}p\left(1-p\right)\frac{{\left(a-b\right)}^{2}}{\max\{a,b\}}\leq d_{1-p}\left(a,b\right)\leq \frac{1}{2}p\left(1-p\right)\frac{{\left(a-b\right)}^{2}}{\min\{a,b\}}$$
  for a,b>0 and 0≤p≤1. By summing up these inequalities, we proved the inequality of the statement:

□

**Remark** **1.** *(i) From the proof of Theorem 3, we obtain $A\left(a,b\right)-H_{p}\left(a,b\right)=\frac{d_{p}\left(a,b\right)+d_{1-p}\left(a,b\right)}{2}$, we deduce an estimation for the Heinz mean:*

(16) $$A\left(a,b\right)-\frac{1}{2}p\left(1-p\right)\frac{{\left(a-b\right)}^{2}}{\min\{a,b\}}\leq H_{p}\left(a,b\right)\leq A\left(a,b\right)-\frac{1}{2}p\left(1-p\right)\frac{{\left(a-b\right)}^{2}}{\max\{a,b\}}.$$

*(ii) Since $d_{p}\left(a,b\right)+d_{1-p}\left(a,b\right)=\left(a^{p}-b^{p}\right)\left(a^{1-p}-b^{1-p}\right)$ and $d_{1-p}\left(a,b\right)\geq 0$, we have $0\leq d_{p}\left(a,b\right)\leq \left(a^{p}-b^{p}\right)\left(a^{1-p}-b^{1-p}\right)$ which is in fact the inequality given by Minguzzi (3).*

**Theorem** **4.** *Let a,b>0 and 0≤p≤1.*

*(i) For 1/2≤p≤1,a≥b or 0≤p≤1/2,a≤b, we have $d_{p}\left(a,b\right)\geq d_{1-p}\left(a,b\right)$.*

*(ii) For 0≤p≤1/2,a≥b or 1/2≤p≤1,a≤b, we have $d_{p}\left(a,b\right)\leq d_{1-p}\left(a,b\right)$.*

**Proof.**  For a=b or p∈{0,1}, we obtain equality in the relations from the statement. Thus, we assume a≠b and $p\in \left(0,1\right)$. However, we have:

$$\begin{array}{ccc} d_{p}\left(a,b\right)-d_{1-p}\left(a,b\right) & = & \left(2p-1\right)\left(a-b\right)-a^{p}b^{1-p}+a^{1-p}b^{p}\\ & = & b\left(\left(2p-1\right)\left(\frac{a}{b}-1\right)-{\left(\frac{a}{b}\right)}^{p}+{\left(\frac{a}{b}\right)}^{1-p}\right). \end{array}$$

We consider the function f:(0,∞)→R defined by $f\left(t\right)=\left(2p-1\right)\left(t-1\right)-t^{p}+t^{1-p}$. We calculate the derivatives of *f*, thus we have:

$$\begin{array}{ccc} & & \frac{df(t)}{dt}=\left(2p-1\right)-pt^{p-1}+\left(1-p\right)t^{-p},\\ & & \frac{d^{2}f\left(t\right)}{dt^{2}}=\left(1-p\right)pt^{p-2}-p\left(1-p\right)t^{-p-1}=p\left(1-p\right)t^{-p-1}\left(t^{2p-1}-1\right). \end{array}$$

For t>1 and 1/2≤p<1, we have $\frac{d^{2}f\left(t\right)}{dt^{2}}>0$, so, function $\frac{df}{dt}$ is increasing, so we obtain $\frac{df\left(t\right)}{dt}>\frac{df\left(1\right)}{dt}=0$, which implies that function *f* is increasing, so we have f(t)>f(1)=0, which means that $\left(2p-1\right)\left(t-1\right)-t^{p}+t^{1-p}>0$. For t=a/b>1, we find that $d_{p}\left(a,b\right)>d_{1-p}\left(a,b\right)$. For t<1 and 0<p≤1/2, we have $\frac{d^{2}f\left(t\right)}{dt^{2}}>0$, so, function $\frac{df}{dt}$ is increasing, so we obtain $\frac{df\left(t\right)}{dt}<\frac{df\left(1\right)}{dt}=0$, which implies that function *f* is decreasing, so we have f(t)>f(1)=0, which means that $\left(2p-1\right)\left(t-1\right)-t^{p}+t^{1-p}>0$. For t=a/b<1, we find that $d_{p}\left(a,b\right)>d_{1-p}\left(a,b\right)$. In the analogous way, we show the inequality in (ii). □

**Remark** **2.** *From (i) in Theorem 4 for 1/2≤p≤1 and a≥b, we have $d_{p}\left(a,b\right)\geq d_{1-p}\left(a,b\right)$, so we obtain:*

(17) $$\frac{1}{2}p\left(1-p\right)\frac{{\left(a-b\right)}^{2}}{\max\{a,b\}}\leq d_{p}\left(a,b\right),$$

*which is just left hand side of Cartwright–Field inequality:*

$$\frac{1}{2}p\left(1-p\right)\frac{{\left(a-b\right)}^{2}}{\max\{a,b\}}\leq d_{p}\left(a,b\right)\leq \frac{1}{2}p\left(1-p\right)\frac{{\left(a-b\right)}^{2}}{\min\{a,b\}},\;\;\left(a,b>0,\;\;0\leq p\leq 1\right).$$

*Therefore, it is quite natural to consider the following inequality:*

$$d_{p}\left(a,b\right)\geq \frac{1}{2}\left\{\frac{1}{2}p\left(1-p\right)\frac{{\left(a-b\right)}^{2}}{\max\{a,b\}}+\frac{1}{2}p\left(1-p\right)\frac{{\left(a-b\right)}^{2}}{\min\{a,b\}}\right\}=\frac{1}{4}p\left(1-p\right){\left(a-b\right)}^{2}\frac{a+b}{ab}$$

*whether it holds or not for a general case a,b>0 and 0≤p≤1. However, this inequality does not hold in general. We set the function:*

$$h_{p}\left(t\right)=pt+1-p-t^{p}-\frac{p(1-p)}{4}\frac{{\left(t-1\right)}^{2}\left(t+1\right)}{t},\;\;\;\;\left(t>0,\;\;\;\;0\leq p\leq 1\right).$$

*Then, we have $h_{0.1}\left(0.3\right)≃-0.00434315$, $h_{0.1}\left(0.6\right)≃0.000199783$ and also $h_{0.9}\left(1.8\right)≃0.000352199$, $h_{0.9}\left(2.6\right)≃-0.00282073$.*

**Theorem** **5.** *For a,b≥1 and 0≤p≤1, we have:*

(18) $$\frac{1}{2}p\left(1-p\right)\frac{{\left(a-b\right)}^{2}}{\max\{a,b\}}\leq \frac{1}{2}E_{p}\left(a,b\right)\leq d_{p}\left(a,b\right),$$

*where $E_{p}\left(a,b\right)≔\min\left\{\frac{p\left(a-b\right)\left(a^{1-p}-b^{1-p}\right)}{{\left(\max\{a,b\}\right)}^{1-p}},\frac{\left(1-p\right)\left(a-b\right)\left(a^{p}-b^{p}\right)}{{\left(\max\{a,b\}\right)}^{p}}\right\}=E_{1-p}\left(a,b\right)$.*

**Proof.**  For p=0 or p=1 or a=b, we have equality. We assume a≠b and 0<p<1. If b<a, then using Theorem 2, we have:

$$\frac{p\left(a-b\right)\left(a^{1-p}-b^{1-p}\right)}{2a^{1-p}}\leq d_{p}\left(a,b\right).$$

Using the Lagrange theorem, we obtain $a^{1-p}-b^{1-p}=\left(1-p\right)\left(a-b\right)\phi^{-p}$, where b<ϕ<a. For b≥1, we deduce $a^{1-p}-b^{1-p}\geq \left(1-p\right)\left(a-b\right)a^{-p}$, which means that $\frac{1}{2}p\left(1-p\right)\frac{{\left(b-a\right)}^{2}}{a}\leq \frac{p\left(a-b\right)\left(a^{1-p}-b^{1-p}\right)}{2a^{1-p}}$. If b>a and we replace *p* by 1−p, then Theorem 2 implies:

(19) $$\frac{\left(1-p\right)\left(a-b\right)\left(a^{p}-b^{p}\right)}{2b^{p}}\leq d_{p}\left(a,b\right).$$

Using the Lagrange theorem, we obtain $b^{p}-a^{p}=p\left(b-a\right)\theta^{p-1}$, where a<θ<b. For a≥1, we deduce $b^{p}-a^{p}\geq p\left(b-a\right)b^{p-1}$, which means that $\frac{1}{2}p\left(1-p\right)\frac{{\left(b-a\right)}^{2}}{b}\leq \frac{\left(1-p\right)\left(a-b\right)\left(a^{p}-b^{p}\right)}{2b^{p}}$. Taking into account the above considerations, we prove the statement. □

**Corollary** **1.** *For 0<a,b≤1 and 0≤p≤1, we have:*

(20) $$\frac{1}{2}p\left(1-p\right)\frac{{\left(a-b\right)}^{2}}{\max\{a,b\}}\leq \frac{ab}{2}E_{p}\left(\frac{1}{a},\frac{1}{b}\right)\leq d_{p}\left(a,b\right),$$

*where $E_{\cdot }\left(\cdot ,\cdot \right)$ is given in Theorem 5.*

**Proof.**  For p=0 or p=1 or a=b, we have the equality. We assume a≠b and 0<p<1. If in inequality (18), we replace a,b≤1 by $\frac{1}{a},\frac{1}{b}\geq 1$, we deduce:

$$\frac{1}{2}p\left(1-p\right)\frac{{\left(a-b\right)}^{2}}{ab\max\{a,b\}}\leq \frac{1}{2}E_{p}\left(\frac{1}{a},\frac{1}{b}\right)\leq d_{p}\left(\frac{1}{a},\frac{1}{b}\right)=\frac{1}{ab}d_{p}\left(a,b\right).$$

Consequently, we prove the inequalities of the statement. □

**Theorem** **6.** *For a,b>0 and 0≤p≤1, we have:*

(21) $$d_{p}\left(a,b\right)\leq \left(1-p\right)\frac{{\left(a-b\right)}^{2}}{b}.$$

**Proof.**  For p=0 or p=1 or a=b, we have equality in the relation from the statement. We assume a≠b and 0<p<1. We consider function f:(0,∞)→R defined by $f\left(t\right)=1-t^{p-1}-\left(1-p\right)\left(t-1\right)$, p∈[0,1]. For t∈(0,1], we have $\frac{df(t)}{dt}=\left(1-p\right)\left(t^{p-2}-1\right)\geq 0$, which implies that *f* is increasing, so we deduce f(t)≤f(1)=0. For t∈[1,∞), we have $\frac{df(t)}{dt}\leq 0$, which implies that *f* is decreasing, so we obtain f(t)≤f(1)=0. Therefore, we find the following inequality:

$$1-t^{p-1}\leq \left(1-p\right)\left(t-1\right).$$

Multiplying the above inequality by t>0, we have:

$$t-t^{p}\leq \left(1-p\right)\left(t^{2}-t\right),$$

which is equivalent to the inequality:

$$pt+\left(1-p\right)-t^{p}\leq \left(1-p\right){\left(t-1\right)}^{2},$$

for all t>0 and p∈[0,1]. Therefore, if we take $t=\frac{a}{b}$ in the above inequality and after some calculations, we deduce the inequality of the statement. □

**Corollary** **2.** *For a,b>0 and 0≤p≤1, we have:*

(22) $$d_{p}\left(a,b\right)+d_{1-p}\left(a,b\right)\leq \left(1-p\right)\frac{{\left(a-b\right)}^{2}\left(a+b\right)}{ab}.$$

**Proof.**  For p=0 or p=1 or a=b, we have the equality. We assume a≠b and 0<p<1. If in inequality (21), we exchange *a* with *b*, we deduce:

$$d_{p}\left(b,a\right)\leq \left(1-p\right)\frac{{\left(a-b\right)}^{2}}{a}.$$

However, $d_{p}\left(b,a\right)=d_{1-p}\left(a,b\right)$, so we have:

$$d_{p}\left(a,b\right)+d_{1-p}\left(a,b\right)\leq \left(1-p\right)\frac{{\left(a-b\right)}^{2}}{b}+\left(1-p\right)\frac{{\left(a-b\right)}^{2}}{a}=\left(1-p\right)\frac{{\left(a-b\right)}^{2}\left(a+b\right)}{ab}.$$

Consequently, we prove the inequality of the statement. □

## 3. Applications to Some Divergences

The Tsallis divergence (e.g., [19,20]) is defined for two probability distributions $p≔\{p_{1},\cdots ,p_{n}\}$ and $r≔\{r_{1},\cdots ,r_{n}\}$ with $p_{j}>0$ and $r_{j}>0$ for all j=1,⋯,n as

$$D_{q}^{T}\left(p|r\right)≔\sum_{j=1}^{n}\frac{p_{j}-p_{j}^{q}r_{j}^{1-q}}{1-q},\;\;\left(q>0,\;\;q\neq 1\right).$$

The Rényi divergence (e.g., [21]) is also denoted by

$$D_{q}^{R}\left(p|r\right)≔\frac{1}{q-1}\log\left(\sum_{j=1}^{n}p_{j}^{q}r_{j}^{1-q}\right).$$

We see in (e.g., [22]) that:

(23) $$D_{q}^{R}\left(p|r\right)=\frac{1}{q-1}\log\left(1+\left(q-1\right)D_{q}^{T}\left(p|r\right)\right).$$

It is also known that:

$$\lim_{q\to 1}D_{q}^{T}\left(p|r\right)=\lim_{q\to 1}D_{q}^{R}\left(p|r\right)=\sum_{j=1}^{n}p_{j}\log\frac{p_{j}}{r_{j}}≕D\left(p|r\right),$$

where D(p|r) is the standard divergence (KL information, reltative entropy). The Jeffreys divergence (see [22,23]) is defined by $J_{1}\left(p|r\right)≔D\left(p|r\right)+D\left(r|p\right)$ and the Jensen–Shannon divergence [15,16] is defined by

$$JS_{1}\left(p|r\right)≔\frac{1}{2}D\left(p|\frac{p+r}{2}\right)+\frac{1}{2}D\left(r|\frac{p+r}{2}\right).$$

In [24], the Jeffreys and the Jensen–Shannon divergence are extended to biparametric forms. In [23], Furuichi and Mitroi generalizes these divergences to the Jeffreys–Tsallis divergence, which is given by $J_{q}\left(p|r\right)≔D_{q}^{T}\left(p|r\right)+D_{q}^{T}\left(r|p\right)$ and to the Jensen–Shannon–Tsallis divergence, which is defined as

$$JS_{q}\left(p|r\right)≔\frac{1}{2}D_{q}^{T}\left(p|\frac{p+r}{2}\right)+\frac{1}{2}D_{q}^{T}\left(r|\frac{p+r}{2}\right).$$

Several properties of divergences can be extended in the operator theory [25].

For the Tsallis divergence, we have the following relations.

**Theorem** **7.** *For two probability distributions $p≔\{p_{1},\cdots ,p_{n}\}$ and $r≔\{r_{1},\cdots ,r_{n}\}$ with $p_{j}>0$ and $r_{j}>0$ for all j=1,⋯,n, we have:*

(24) $$q\sum_{j=1}^{n}\frac{{\left(p_{j}-r_{j}\right)}^{2}}{\max\{p_{j},r_{j}\}}\leq J_{q}\left(p|r\right)\leq q\sum_{j=1}^{n}\frac{{\left(p_{j}-r_{j}\right)}^{2}}{\min\{p_{j},r_{j}\}},\;\;\left(0<q<1\right).$$

**Proof.**  From the definition of the Tsallis divergence, we deduce the equality:

$$J_{q}\left(p|r\right)=\sum_{j=1}^{n}\frac{p_{j}+r_{j}-p_{j}^{q}r_{j}^{1-q}-p_{j}^{1-q}r_{j}^{q}}{1-q}=\frac{1}{1-q}\sum_{j=1}^{n}\left\{d_{q}\left(p_{j},r_{j}\right)+d_{1-q}\left(p_{j},r_{j}\right)\right\},$$

where $d_{\cdot }\left(\cdot ,\cdot \right)$ is defined in (6). Applying Theorem 3, we obtain:

$$q\left(1-q\right)\sum_{j=1}^{n}\frac{{\left(p_{j}-r_{j}\right)}^{2}}{\max\{p_{j},r_{j}\}}\leq \sum_{j=1}^{n}\left\{d_{q}\left(p_{j},r_{j}\right)+d\left(p_{j},r_{j}\right)\right\}\leq q\left(1-q\right)\sum_{j=1}^{n}\frac{{\left(p_{j}-r_{j}\right)}^{2}}{\min\{p_{j},r_{j}\}}$$

and combining with the above equality, we deduce the inequalities (24). □

**Remark** **3.** *(i) In the limit of q→1 in* (24)*, we then obtain:*

$$\sum_{j=1}^{n}\frac{{\left(p_{j}-r_{j}\right)}^{2}}{\max\{p_{j},r_{j}\}}\leq J_{1}\left(p|r\right)\leq \sum_{j=1}^{n}\frac{{\left(p_{j}-r_{j}\right)}^{2}}{\min\{p_{j},r_{j}\}}$$

*for the standard divergence.*

*(ii)* *From (23), we have:*

$$\begin{array}{ccc} 2+\left(q-1\right)\left\{D_{q}^{T}\left(p|r\right)+D_{q}^{T}\left(r|p\right)\right\} & = & \exp\left(\left(q-1\right)D_{q}^{R}\left(p|r\right)\right)+\exp\left(\left(q-1\right)D_{q}^{R}\left(r|p\right)\right)\\ & \geq & 2+\left(q-1\right)\left\{D_{q}^{R}\left(p|r\right)+D_{q}^{R}\left(r|p\right)\right\}, \end{array}$$

*where we used the inequality $e^{x}\geq x+1$ for all x∈R. Thus, we deduce the inequalities:*

(25) $$D_{q}^{T}\left(p|r\right)+D_{q}^{T}\left(r|p\right)\leq D_{q}^{R}\left(p|r\right)+D_{q}^{R}\left(r|p\right),\;\;\left(0<q<1\right)$$

*and:*

$$D_{q}^{T}\left(p|r\right)+D_{q}^{T}\left(r|p\right)\geq D_{q}^{R}\left(p|r\right)+D_{q}^{R}\left(r|p\right),\;\;\left(q>1\right).$$

*Combining (25) with Theorem 7, we therefore have the following result for the Rényi divergence:*

$$q\sum_{j=1}^{n}\frac{{\left(p_{j}-r_{j}\right)}^{2}}{\max\{p_{j},r_{j}\}}\leq D_{q}^{R}\left(p|r\right)+D_{q}^{R}\left(r|p\right),\;\;\left(0<q<1\right).$$

We give the relation between the Jeffreys–Tsallis divergence and the Jensen–Shannon–Tsallis divergence:

**Theorem** **8.** *For two probability distributions $p≔\{p_{1},\cdots ,p_{n}\}$ and $r≔\{r_{1},\cdots ,r_{n}\}$ with $p_{j}>0$ and $r_{j}>0$ for all j=1,⋯,n, we have:*

(26) $$JS_{q}\left(p|r\right)\leq \frac{1}{4}J_{q}\left(p|r\right),$$

*where q≥0 with q≠1.*

**Proof.**  We consider the function g:(0,∞)→R defined by $g\left(t\right)=t^{1-q}$, which is concave for q∈[0,1). Therefore, we have ${\left(\frac{p_{j}+r_{j}}{2}\right)}^{1-q}\geq \frac{p_{j}^{1-q}+r_{j}^{1-q}}{2}$, which implies the following inequalities:

$$p_{j}-p_{j}^{q}{\left(\frac{p_{j}+r_{j}}{2}\right)}^{1-q}\leq \frac{p_{j}-p_{j}^{q}r_{j}^{1-q}}{2},r_{j}-r_{j}^{q}{\left(\frac{p_{j}+r_{j}}{2}\right)}^{1-q}\leq \frac{r_{j}-r_{j}^{q}p_{j}^{1-q}}{2},$$

From the definition of the Tsallis divergence, we deduce the inequality:

$$D_{q}^{T}\left(p|\frac{p+r}{2}\right)+D_{q}^{T}\left(r|\frac{p+r}{2}\right)\leq \frac{1}{2}\left(D_{q}^{T}\left(p|r\right)+D_{q}^{T}\left(r|p\right)\right),$$

which is equivalent to the relation of the statement. For the case of q>1, the function $g\left(t\right)=t^{1-q}$ is convex in t>0. Similarly, we have the statement, taking into account that 1−q<0. □

**Remark** **4.** *In the limit of q→1 in (26), we then obtain:*

$$JS_{1}\left(p|r\right)\leq \frac{1}{4}J_{1}\left(p|r\right).$$

We give the bounds on the Jeffreys–Tsallis divergence by using the refined Young inequality given in Theorem 1. In [26], we found the *Battacharyya coefficient* defined as

$$B\left(p|r\right)≔\sum_{j=1}^{n}\sqrt{p_{j}r_{j}},$$

which is a measure of the amount of overlapping between two distributions. This can be expressed in terms of the Hellinger distance between the probability distributions $p≔\{p_{1},\cdots ,p_{n}\}$ and $r≔\{r_{1},\cdots ,r_{n}\}$, which is given by

$$B\left(p|r\right)=1-h^{2}\left(p|r\right),$$

where the *Hellinger distance* ([26,27]) is a metric distance and defined by

$$h\left(p|r\right)≔\frac{1}{\sqrt{2}}\sqrt{\sum_{j=1}^{n}{\left(\sqrt{p_{j}}-\sqrt{r_{j}}\right)}^{2}}.$$

**Theorem** **9.** *For two probability distributions $p≔\{p_{1},\cdots ,p_{n}\}$ and $r≔\{r_{1},\cdots ,r_{n}\}$ with $p_{j}>0$ and $r_{j}>0$ for all j=1,⋯,n, and 0≤q<1, we have:*

(27) $$\begin{array}{c} \frac{4r}{1-q}h^{2}\left(p|r\right)+\frac{2A\left(q\right)}{1-q}\sum_{j=1}^{n}p_{j}r_{j}\cdot \log^{2}\left(\frac{p_{j}}{r_{j}}\right)\leq J_{q}\left(p|r\right)\\ \leq \frac{4\left(1-r\right)}{1-q}h^{2}\left(p|r\right)+\frac{2B\left(q\right)}{1-q}\sum_{j=1}^{n}p_{j}r_{j}\cdot \log^{2}\left(\frac{p_{j}}{r_{j}}\right). \end{array}$$

*where $r=\min\left\{q,1-q\right\}$ and $A\left(q\right)=\frac{q\left(1-q\right)}{2}-\frac{r}{4},B\left(q\right)=\frac{q\left(1-q\right)}{2}-\frac{1-r}{4}$.*

**Proof.**  For q=0, we obtain the equality. Now, we consider 0<q<1. Using Theorem 1 for $a=p_{j}<1$ and $b=r_{j}<1$, j∈{1,2,...,n}, we deduce:

$$r{\left({\sqrt{p}}_{j}-{\sqrt{r}}_{j}\right)}^{2}+A\left(q\right)p_{j}r_{j}\cdot \log^{2}\left(\frac{p_{j}}{r_{j}}\right)\leq d_{q}\left(p_{j},r_{j}\right)$$

$$\leq \left(1-r\right){\left({\sqrt{p}}_{j}-{\sqrt{r}}_{j}\right)}^{2}+B\left(q\right)p_{j}r_{j}\cdot \log^{2}\left(\frac{p_{j}}{r_{j}}\right),$$

where $r=\min\left\{q,1-q\right\}$. If we replace *q* by 1−q and taking into account that $A\left(q\right)=A\left(1-q\right)$ and $B\left(q\right)=B\left(1-q\right)$, then we have:

$$2r{\left({\sqrt{p}}_{j}-{\sqrt{r}}_{j}\right)}^{2}+2A\left(q\right)p_{j}r_{j}\cdot \log^{2}\left(\frac{p_{j}}{r_{j}}\right)\leq d_{q}\left(p_{j},r_{j}\right)+d_{1-q}\left(p_{j},r_{j}\right)$$

$$\leq 2\left(1-r\right){\left({\sqrt{p}}_{j}-{\sqrt{r}}_{j}\right)}^{2}+2B\left(q\right)p_{j}r_{j}\cdot \log^{2}\left(\frac{p_{j}}{r_{j}}\right).$$

Taking the sum on j=1,2,⋯,n, we find the inequalities:

$$2r\sum_{j=1}^{n}{\left({\sqrt{p}}_{j}-{\sqrt{r}}_{j}\right)}^{2}+2A\left(q\right)\sum_{j=1}^{n}p_{j}r_{j}\cdot \log^{2}\left(\frac{p_{j}}{r_{j}}\right)\leq \sum_{j=1}^{n}\left(d_{q}\left(p_{j},r_{j}\right)+d_{1-q}\left(p_{j},r_{j}\right)\right)$$

$$=\left(1-q\right)\left(D_{q}^{T}\left(p|r\right)+D_{q}^{T}\left(r|p\right)\right)\leq 2\left(1-r\right)\sum_{j=1}^{n}{\left({\sqrt{p}}_{j}-{\sqrt{r}}_{j}\right)}^{2}+2B\left(q\right)\sum_{j=1}^{n}p_{j}r_{j}\cdot \log^{2}\left(\frac{p_{j}}{r_{j}}\right),$$

which is equivalent to the inequalities in the statement. □

**Remark** **5.** *In the limit of q→1 in* (27)*, we then obtain:*

$$4h^{2}\left(p|r\right)+\frac{1}{2}\sum_{j=1}^{n}p_{j}r_{j}\cdot \log^{2}\left(\frac{p_{j}}{r_{j}}\right)\leq J_{1}\left(p|r\right),$$

*since $\lim_{q\to 1}\frac{r}{1-q}=1$, $\lim_{q\to 1}\frac{A(q)}{1-q}=\frac{1}{4}$ and $\lim_{q\to 1}\frac{1-r}{1-q}=\infty$.*

We give the further bounds on the Jeffreys–Tsallis divergence by the use of Theorem 5 and Corollary 2:

**Theorem** **10.** *For two probability distributions $p≔\{p_{1},\cdots ,p_{n}\}$ and $r≔\{r_{1},\cdots ,r_{n}\}$ with $p_{j}>0$ and $r_{j}>0$ for all j=1,⋯,n, and 0≤q<1, we have:*

(28) $$q\sum_{j=1}^{n}\frac{{\left(p_{j}-r_{j}\right)}^{2}}{\max\{p_{j},r_{j}\}}\leq \frac{1}{1-q}\sum_{j=1}^{n}p_{j}r_{j}E_{q}\left(\frac{1}{p_{j}},\frac{1}{r_{j}}\right)\leq J_{q}\left(p|r\right)\leq \sum_{j=1}^{n}\frac{{\left(p_{j}-r_{j}\right)}^{2}\left(p_{j}+r_{j}\right)}{p_{j}r_{j}},$$

*where $E_{\cdot }\left(\cdot ,\cdot \right)$ is given in Theorem 5.*

**Proof.**  Putting $a≔p_{j}$, $b≔r_{j}$ and p≔q in (20), we deduce:

$$\frac{1}{2}q\left(1-q\right)\frac{{\left(p_{j}-r_{j}\right)}^{2}}{\max\{p_{j},r_{j}\}}\leq \frac{p_{j}r_{j}}{2}E_{q}\left(\frac{1}{p_{j}},\frac{1}{r_{j}}\right)\leq d_{q}\left(p_{j},r_{j}\right),$$

and:

$$\frac{1}{2}q\left(1-q\right)\frac{{\left(p_{j}-r_{j}\right)}^{2}}{\max\{p_{j},r_{j}\}}\leq \frac{p_{j}r_{j}}{2}E_{1-q}\left(\frac{1}{p_{j}},\frac{1}{r_{j}}\right)\leq d_{1-q}\left(p_{j},r_{j}\right).$$

Taking into account that:

$$E_{q}\left(\frac{1}{p_{j}},\frac{1}{r_{j}}\right)=E_{1-q}\left(\frac{1}{p_{j}},\frac{1}{r_{j}}\right),$$

and by taking the sum on j=1,2,⋯,n, we have:

$$q\left(1-q\right)\sum_{j=1}^{n}\frac{{\left(p_{j}-r_{j}\right)}^{2}}{\max\{p_{j},r_{j}\}}\leq \sum_{j=1}^{n}p_{j}r_{j}E_{q}\left(\frac{1}{p_{j}},\frac{1}{r_{j}}\right)\leq \sum_{j=1}^{n}\left(d_{q}\left(p_{j},r_{j}\right)+d_{1-q}\left(p_{j},r_{j}\right)\right)$$

we prove the lower bounds of $J_{q}\left(p|r\right)$. To prove the upper bound of $J_{q}\left(p|r\right)$, we put $a≔p_{j}$, $b≔r_{j}$ and p≔q in inequality (22). Then, we deduce:

$$d_{q}\left(p_{j},r_{j}\right)+d_{1-q}\left(p_{j},r_{j}\right)\leq \left(1-q\right)\frac{{\left(p_{j}-r_{j}\right)}^{2}\left(p_{j}+r_{j}\right)}{p_{j}r_{j}}.$$

By taking the sum on j=1,2,⋯,n, we find:

$$\sum_{j=1}^{n}\left(d_{q}\left(p_{j},r_{j}\right)+d_{1-q}\left(p_{j},r_{j}\right)\right)\leq \left(1-q\right)\sum_{j=1}^{n}\frac{{\left(p_{j}-r_{j}\right)}^{2}\left(p_{j}+r_{j}\right)}{p_{j}r_{j}}.$$

Consequently, we prove the inequalities of the statement. □

We also give the further bounds on the Jeffreys–Tsallis divergence by the use of Cartwright–Field inequality given in (7).

**Theorem** **11.** *For two probability distributions $p≔\{p_{1},\cdots ,p_{n}\}$ and $r≔\{r_{1},\cdots ,r_{n}\}$ with $p_{j}>0$ and $r_{j}>0$ for all j=1,⋯,n, and 0≤q<1, we have:*

(29) $$\begin{array}{c} \frac{q}{8}\sum_{j=1}^{n}{\left(p_{j}-r_{j}\right)}^{2}\left(\frac{1}{p_{j}+\max\left\{p_{j},r_{j}\right\}}+\frac{1}{r_{j}+\max\left\{p_{j},r_{j}\right\}}\right)\\ \leq JS_{q}\left(p|r\right)\leq \frac{q}{8}\sum_{j=1}^{n}{\left(p_{j}-r_{j}\right)}^{2}\left(\frac{1}{p_{j}+\min\left\{p_{j},r_{j}\right\}}+\frac{1}{r_{j}+\min\left\{p_{j},r_{j}\right\}}\right) \end{array}$$

**Proof.**  For q=0, we have the equality. We assume 0<q<1. By direct calculations, we have:

$$\begin{array}{ccc} & & JS_{q}\left(p|r\right)=\frac{1}{2}D_{q}^{T}\left(p|\frac{p+r}{2}\right)+\frac{1}{2}D_{q}^{T}\left(r|\frac{p+r}{2}\right)\\ & & =\frac{1}{2(1-q)}\sum_{j=1}^{n}\left\{p_{j}-p_{j}^{q}{\left(\frac{p_{j}+r_{j}}{2}\right)}^{1-q}+r_{j}-r_{j}^{q}{\left(\frac{p_{j}+r_{j}}{2}\right)}^{1-q}\right\}\\ & & =\frac{1}{2(1-q)}\sum_{j=1}^{n}\left\{qp_{j}+\left(1-q\right)\frac{p_{j}+r_{j}}{2}-p_{j}^{q}{\left(\frac{p_{j}+r_{j}}{2}\right)}^{1-q}+qr_{j}+\left(1-q\right)\frac{p_{j}+r_{j}}{2}-r_{j}^{q}{\left(\frac{p_{j}+r_{j}}{2}\right)}^{1-q}\right\}\\ & & =\frac{1}{2(1-q)}\sum_{j=1}^{n}\left\{d_{q}\left(p_{j},\frac{p_{j}+r_{j}}{2}\right)+d_{q}\left(r_{j},\frac{p_{j}+r_{j}}{2}\right)\right\}. \end{array}$$

Using inequality (7), we deduce:

$$\frac{q(1-q)}{4}\frac{{\left(p_{j}-r_{j}\right)}^{2}}{p_{j}+\max\left\{p_{j},r_{j}\right\}}\leq d_{q}\left(p_{j},\frac{p_{j}+r_{j}}{2}\right)\leq \frac{q(1-q)}{4}\frac{{\left(p_{j}-r_{j}\right)}^{2}}{p_{j}+\min\left\{p_{j},r_{j}\right\}}$$

and:

$$\frac{q(1-q)}{4}\frac{{\left(p_{j}-r_{j}\right)}^{2}}{r_{j}+\max\left\{p_{j},r_{j}\right\}}\leq d_{q}\left(r_{j},\frac{p_{j}+r_{j}}{2}\right)\leq \frac{q(1-q)}{4}\frac{{\left(p_{j}-r_{j}\right)}^{2}}{r_{j}+\min\left\{p_{j},r_{j}\right\}}.$$

From the above inequalities, we have the statement, by summing on j=1,2,⋯,n. □

It is quite natural to extend the Jensen–Shannon–Tsallis divergence to the following form:

$$JS_{q}^{v}\left(p|r\right)≔vD_{q}^{T}\left(p|vp+\left(1-v\right)r\right)+\left(1-v\right)D_{q}^{T}\left(r|vp+\left(1-v\right)r\right),$$

where 0≤v≤1,q>0,q≠1. We call this the *v*-weighted Jensen–Shannon–Tsallis divergence. For v=1/2, we find that $JS_{q}^{1/2}\left(p|r\right)=JS_{q}\left(p|r\right)$ which is the Jensen–Shannon–Tsallis divergence. For this quantity $JS_{q}^{v}\left(p|r\right)$, we can obtain the following result in a way similar to the proof of the Theorem 11.

**Proposition** **1.** *For two probability distributions $p≔\{p_{1},\cdots ,p_{n}\}$ and $r≔\{r_{1},\cdots ,r_{n}\}$ with $p_{j}>0$ and $r_{j}>0$ for all j=1,⋯,n, 0≤q<1 and 0≤v≤1, we have:*

$$\begin{array}{ccc} & & \frac{qv(1-v)}{2}\sum_{j=1}^{n}{\left(p_{j}-r_{j}\right)}^{2}\left(\frac{1-v}{vp_{j}+\left(1-v\right)\max\left\{p_{j},r_{j}\right\}}+\frac{v}{\left(1-v\right)r_{j}+v\max\left\{p_{j},r_{j}\right\}}\right)\\ & & \leq JS_{q}^{v}\left(p|r\right)\\ & & \leq \frac{qv(1-v)}{2}\sum_{j=1}^{n}{\left(p_{j}-r_{j}\right)}^{2}\left(\frac{1-v}{vp_{j}+\left(1-v\right)\min\left\{p_{j},r_{j}\right\}}+\frac{v}{\left(1-v\right)r_{j}+v\min\left\{p_{j},r_{j}\right\}}\right). \end{array}$$

**Proof.**  We calculate as

$$\begin{array}{ccc} & & JS_{q}^{v}\left(p|r\right)=\frac{v}{1-q}\sum_{j=1}^{n}\left\{p_{j}-p_{j}^{q}{\left(vp_{j}+\left(1-v\right)r_{j}\right)}^{1-q}\right\}+\frac{1-v}{1-q}\sum_{j=1}^{n}\left\{r_{j}-r_{j}^{q}{\left(vp_{j}+\left(1-v\right)r_{j}\right)}^{1-q}\right\}\\ & & =\frac{1}{1-q}\sum_{j=1}^{n}\left\{vp_{j}+\left(1-v\right)r_{j}-vp_{j}^{q}{\left(vp_{j}+\left(1-v\right)r_{j}\right)}^{1-q}-\left(1-v\right)r_{j}^{q}{\left(vp_{j}+\left(1-v\right)r_{j}\right)}^{1-q}\right\}\\ & & =\frac{v}{1-q}\sum_{j=1}^{n}\left\{qp_{j}+\left(1-q\right)\left(vp_{j}+\left(1-v\right)r_{j}\right)-p_{j}^{q}{\left(vp_{j}+\left(1-v\right)r_{j}\right)}^{1-q}\right\}\\ & & +\frac{1-v}{1-q}\sum_{j=1}^{n}\left\{qr_{j}+\left(1-q\right)\left(vp_{j}+\left(1-v\right)r_{j}\right)-r_{j}^{q}{\left(vp_{j}+\left(1-v\right)r_{j}\right)}^{1-q}\right\}\\ & & =\frac{1}{1-q}\sum_{j=1}^{n}\left\{vd_{q}\left(p_{j},vp_{j}+\left(1-v\right)r_{j}\right)+\left(1-v\right)d_{q}\left(r_{j},vp_{j}+\left(1-v\right)r_{j}\right)\right\}. \end{array}$$

Using inequality (7), we deduce:

$$\frac{q(1-q)}{2}\frac{{\left(1-v\right)}^{2}{\left(p_{j}-r_{j}\right)}^{2}}{vp_{j}+\left(1-v\right)\max\left\{p_{j},r_{j}\right\}}\leq d_{q}\left(p_{j},vp_{j}+\left(1-v\right)r_{j}\right)$$

$$\leq \frac{q(1-q)}{2}\frac{{\left(1-v\right)}^{2}{\left(p_{j}-r_{j}\right)}^{2}}{vp_{j}+\left(1-v\right)\min\left\{p_{j},r_{j}\right\}}$$

and:

$$\frac{q(1-q)}{2}\frac{v^{2}{\left(p_{j}-r_{j}\right)}^{2}}{\left(1-v\right)r_{j}+v\max\left\{p_{j},r_{j}\right\}}\leq d_{q}\left(r_{j},vp_{j}+\left(1-v\right)r_{j}\right)$$

$$\leq \frac{q(1-q)}{2}\frac{v^{2}{\left(p_{j}-r_{j}\right)}^{2}}{\left(1-v\right)r_{j}+v\min\left\{p_{j},r_{j}\right\}}.$$

Multiplying *v* and 1−v by the above inequalities, respectively, and then taking the sum on j=1,2,⋯,n, we obtain the statement. □

## 4. Conclusions

We obtained new inequalities which improve classical Young inequality by analytical calculations with known inequalities. We also obtained some bounds on the Jeffreys–Tsallis divergence and the Jensen–Shannon–Tsallis divergence. At this point, we do not clearly know whether the obtained bounds will play any role in the information theory. However, if there exists a purpose to find the meaning of the parameter *q* in divergences based on Tsallis divergence, then we may state that almost all theorems (except for Theorem 8) hold for 0≤q<1. In the first author’s previous studies [19,28], some results related to Tsallis divergence (relative entropy) are still true for 0≤q<1, while some results related to Tsallis entropy are still true for q>1. In this paper, we treated the Tsallis type divergence so it is shown that almost all results are true for 0≤q<1. This insight may give a rough meaning of the parameter *q*.

Since our results in Section 3 are based on the inequalities in Section 2, we summarized the tightness for our obtained inequalities in Section 2. The double inequality (12) is a counterpart of the double inequality (9) for a,b∈(0,1]. Therefore, they can not be compared wit each other from the point of view on the tightness, since the conditions are different. The double inequality (12) was used to obtain Theorem 9. The double inequality (15) is essentially a Cartwright–Field inequality in itself, and it was used to obtain Theorem 7 as the first result in Section 3. The results in Theorem 4 are mathematical properties on $d_{p}\left(a,b\right)$. The inequalities given in (18) gave an improvement of the left-hand side in the inequality (7) for the case a,b≥1 and we obtained Theorem 10 by (18). We obtained the upper bound of $d_{p}\left(a,b\right)$ as a counterpart of (18) for a general a,b>0. This is used to prove Corollary 2 which was used to prove Theorem 10. However, we found that the upper bound of $d_{p}\left(a,b\right)+d_{1-p}\left(a,b\right)$ given in (22) is not tighter than the one in (15).

Finally, Theorem 8 can be obtained from the convexity/concavity of the function $t^{1-q}$. This study will be continued in order to obtain much sharper bounds. We extend the Jensen–Shannon–Tsallis divergence to the following:

$$JS_{q}^{v}\left(p|r\right)≔vD_{q}^{T}\left(p|vp+\left(1-v\right)r\right)+\left(1-v\right)D_{q}^{T}\left(r|vp+\left(1-v\right)r\right),\;\;\left(0\leq v\leq 1,\;\;q>0,\;\;q\neq 1\right),$$

and we call this the *v*-weighted Jensen–Shannon–Tsallis divergence. For v=1/2, we find that $JS_{q}^{1/2}\left(p|r\right)=JS_{q}\left(p|r\right)$ which is the Jensen–Shannon–Tsallis divergence. For this quantity, as a information-theoretic divergence measure $JS_{q}^{v}\left(p|r\right)$, we obtained several characterizations.

## Data Availability

Not applicable.
